# Return to sports and clinical outcomes in patients treated for peroneal tendon dislocation: a systematic review

**DOI:** 10.1007/s00167-015-3833-z

**Published:** 2015-10-30

**Authors:** Pim A. D. van Dijk, Arianna L. Gianakos, Gino M. M. J. Kerkhoffs, John G. Kennedy

**Affiliations:** Foot and Ankle Service, Hospital for Special Surgery, 523 East 72nd Street, East River Professional Building, 5th Floor, Rm 507, New York, NY 10021 USA; Department of Orthopedic Surgery, Academic Medical Center, Amsterdam, The Netherlands; Academic Center for Evidence based Sports medicine (ACES), Amsterdam, The Netherlands; Amsterdam Collaboration for Health and Safety in Sports (ACHSS), Amsterdam, The Netherlands

**Keywords:** Peroneal tendon, Fibular tendon, Dislocation, Subluxation, Groove deepening, Retinaculum repair, Return to sports

## Abstract

**Purpose:**

The aim of this study was to determine the outcome following different surgical treatment techniques in the treatment of peroneal tendon dislocation and to establish whether return to sports was achieved universally following the procedures.

**Methods:**

A systematic review and best-evidence synthesis was performed. PubMed and EMBASE were searched for eligible studies. The last search was done in March 2015. Quality assessment of pooled data was performed using a modified Macleod scale and a best-evidence synthesis was performed. In total, 14 studies were included.

**Results:**

Surgical treatment provides improvement in the post-operative AOFAS score (*p* < 0.0001) and high satisfaction rates. The redislocation rate is less than 1.5 % at long-term follow-up. Patients treated with both groove deepening and SPR repair have higher rates of return to sports than patients treated with SPR repair alone (*p* = 0.022).

**Conclusions:**

Surgical treatment of peroneal tendon dislocation provides good outcomes, high satisfaction and a quick return to sports. Rates in return to sports are significantly higher in patients treated with both groove deepening and SPR repair. To optimize treatment, the surgical management should involve increasing the superior peroneal tunnel volume by groove deepening and stabilizing the tendons by SPR repair.

**Level of evidence:**

Level IV, systematic review of level IV studies.

## Introduction

Peroneal tendon dislocation occurs in 0.3–0.5 % of all traumatic ankle events and is often misdiagnosed and therefore underreported [30]. Peroneal tendon dislocation is most prevalent in the athletic population, primarily in sports which require cutting movements including skiing [23], soccer, basketball, ice skating and gymnastics [3]. Patients may report a snapping or popping sensation around the lateral malleolus and complain of significant functional impairment. To provide early return to sports (RTS), optimal treatment is critical. Although many treatment options are described in the literature, consensus on the best treatment algorithm has yet to be established [26].

**Fig. 1:**
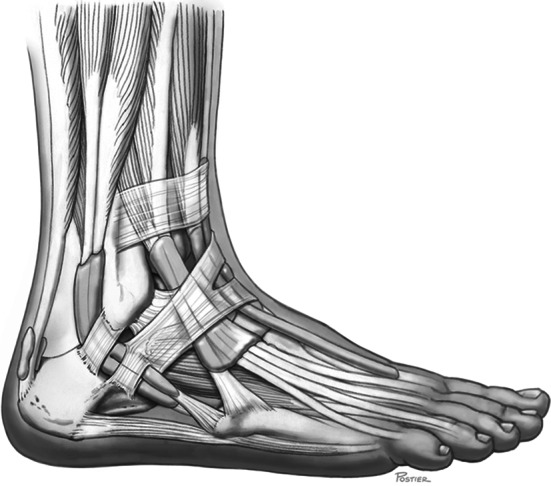
The anatomy of the lateral ankle

Peroneal tendon dislocation typically occurs when the peroneal muscles suddenly eccentrically contract on acute dorsiflexion of the foot, with or without inversion, or during forced dorsiflexion of the everted foot. This can result in a rupture of the superior peroneal retinaculum, allowing the peroneal tendons to dislocate anteriorly over the lateral malleolus. Previous studies have demonstrated that flat or convex retromalleolar grooves may predispose patients to luxation of the peroneal tendons [14, 32]. The presence of a peroneus quartus muscle or a low-lying muscle belly makes individuals also more susceptible for peroneal tendon dislocation [37, 42, 43]. Normal anatomy of the lateral ankle is shown in Fig. 1.

**Fig. 2:**
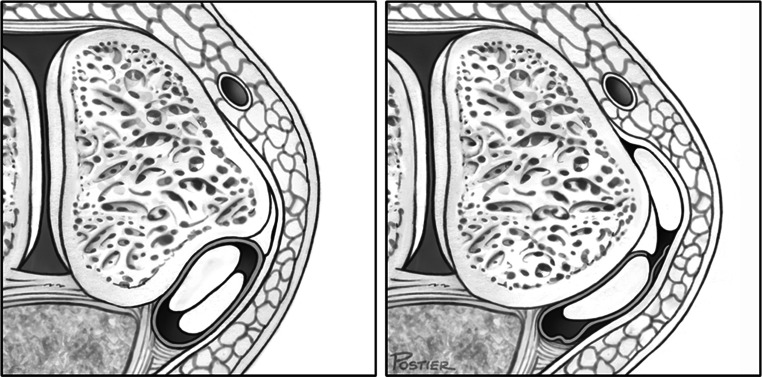
The superior peroneal tunnel: normal anatomy (left) and subluxation of the peroneus longus tendon over the lateral malleolus (right)

**Fig. 3:**
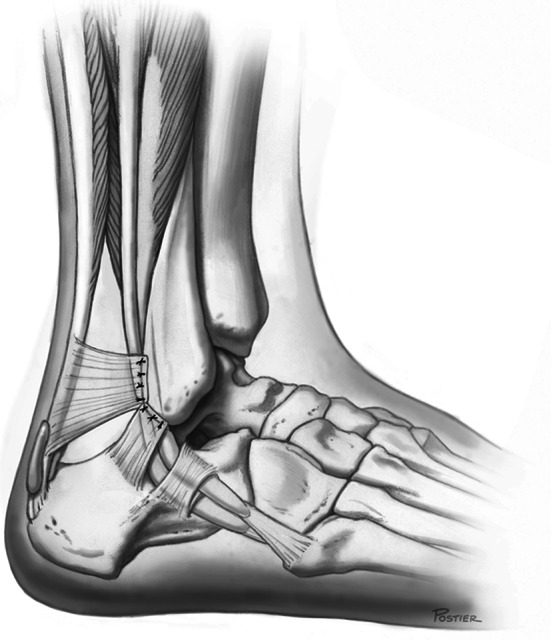
The lateral ankle after repair of the superior peroneal retinaculum

**Fig. 4:**
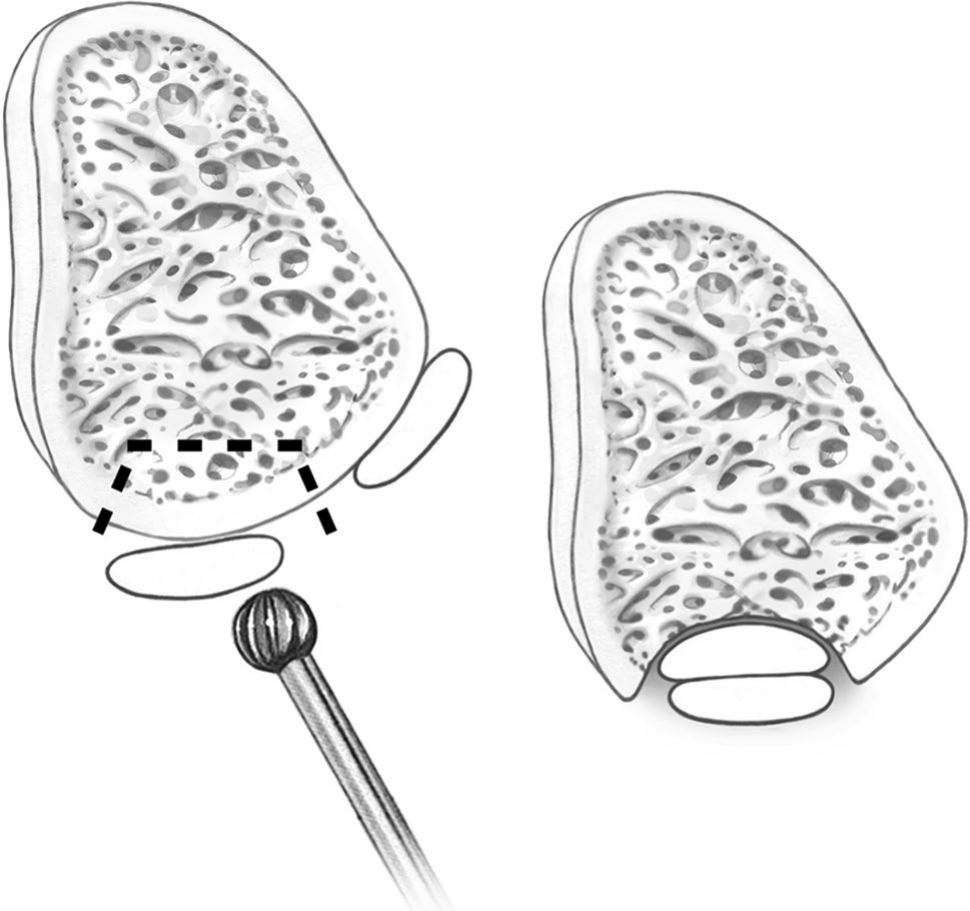
Groove deepening of the retromalleolar groove

Conservative treatment may be attempted in patients with acute dislocation, but the literature reports a failure rate of 50–76 % [8, 9]. Surgical procedures have become the preferred method of treatment, especially in young, active people and athletes [24]. More than 20 surgical techniques have been recommended for stabilizing the peroneal tendons. These procedures attempt to repair the superior peroneal tunnel, which is formed by the superior peroneal retinaculum (SPR), retromalleolar groove and dorsal intermuscular septum (Fig. 2). The primary treatment strategies can be divided into the following four main categories: (1) repair or replacement of the SPR (Fig. 3) [2, 4, 6, 7, 12, 16, 20, 35], (2) groove deepening of the retromalleolar groove (Fig. 4) [13, 27, 28, 44], (3) bony procedures [21, 43] or (4) rerouting procedures [19, 31, 36]. Most studies utilizing these procedures have demonstrated good-to-excellent outcomes and a high rate of return to sports [13, 27, 28, 31, 36, 44].

Although numerous treatment strategies have been previously described, there is a lack of consensus on how to treat patients diagnosed with peroneal tendon dislocation. In order to evaluate currently used surgical treatment options and to create a treatment strategy for optimal functional outcomes, a review of available evidence is required. The purpose of this systematic review was to (1) determine the outcome after different surgical treatment techniques of peroneal tendon dislocation and (2) compare the rates of return to sports and clinical outcomes in different surgical techniques. It is hypothesized that operative treatment of peroneal tendon dislocation leads to good functional outcomes and allows for return to sports at the pre-injury level with normal peroneal tendon function.

## Material and methods

### Search strategy

Relevant publications were identified by searching PubMed/MEDLINE and the EMBASE electronic database in March of 2015. Three keywords (peroneal, dislocation and treatment) and related synonyms were used. All synonyms were combined with the Boolean command AND and were linked by the Boolean command OR.

### Eligibility criteria

Original studies were included if (1) diagnosis on peroneal subluxation or dislocation was confirmed during surgery, (2)  the AOFAS or return to sports was described, (3) the surgical technique was well described and (4) full texts were available in English. Exclusion criteria were (1) case reports, imaging reviews, surgical technique reports and animal studies, (2) studies with less then 10 participants, (3) studies with a primary purpose other then to report the outcomes of a peroneal tendon dislocation treatment and (4) studies with a mean follow-up of  less then 6 months.

### Study selection

One author performed the literature search (PAD), and two authors independently reviewed the search results (PAD, AG). The titles and abstracts were reviewed by applying the eligibility criteria, and potential relevant studies were reviewed on full text. The reference lists of included studies were also reviewed and compared with the collected studies to ensure no pertinent studies were omitted.

### Data extraction

Pertinent data from the original articles were extracted using a modified extraction form. Whenever an outcome was reported at more than one point in time during follow-up, values of the last recorded follow-up were used.

### Quality assessment

Quality assessment of the included studies was performed by two authors independently (PAD, AG) using the modified Macleod scale [17]. Included criteria were: published in a peer-reviewed journal, reported gender of included patients, reported inclusion and exclusion criteria, reported concomitant comorbidities, presence of a control group, random allocation to treatment or control, blinded assessment of outcome, reported follow-up and statement of potential conflict of interests. If no consensus was reached, the independent opinion of a third reviewer (JGK) was established.

### Best-evidence synthesis

A modified version of the best-evidence synthesis was used to combine results because of the poor level of evidence and the heterogeneity of outcome measures [34]. The results of the quality of evidence assessments were used to classify the level of evidence [40]. This qualitative analysis was performed with five levels of evidence, based on the quality and results of the included studies:

1. Strong evidence: provided by two or more high-quality studies and by generally consistent findings in all studies (75 % of the studies reported consistent findings).
2. Moderate evidence: provided by one high-quality study and/or three or more low-quality studies and by generally consistent findings in all studies (75 % of the studies reported consistent findings).
3. Limited evidence: provided by two or less low-quality studies.
4. Conflicting evidence: inconsistent findings in multiple studies (less than 75 % of the studies reported consistent findings).
5. No evidence: when no studies could be found.

### Statistical analysis

Independent samples *t* tests were used for comparison of group means in return-to-sports rate and time, and a paired-samples *t* test was used to compare pre-operative and post-operative AOFAS scores. A *p* value of less than 0.05 was considered as statistically significant. Statistical analysis was performed using Stata version 13.0 software (STATA Corp., TX, USA).

## Results

### Search and literature selection

**Fig. 5:**
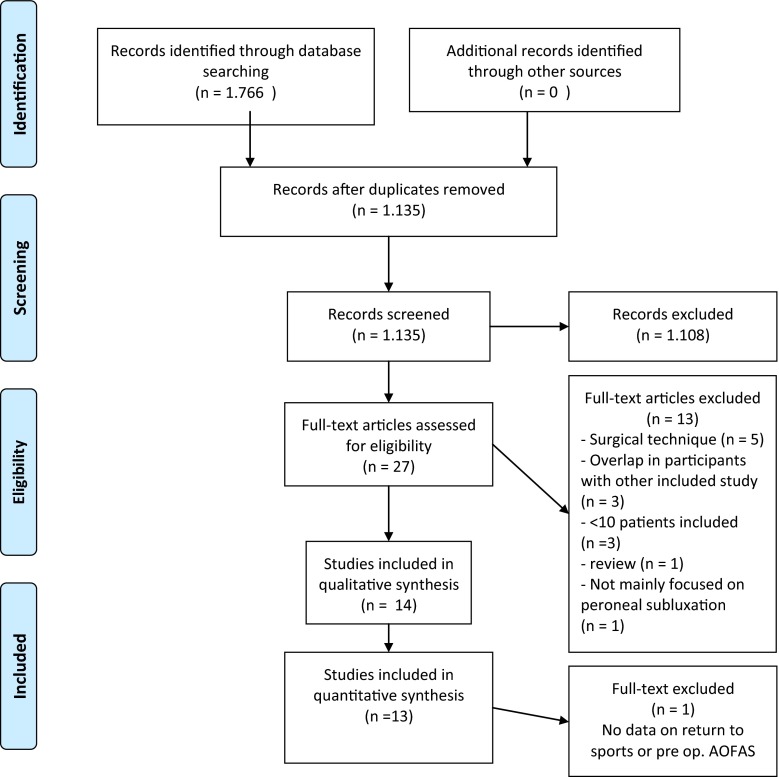
PRISMA flow diagram

The literature search in PubMed/MEDLINE and EMBASE databases yielded 925 and 841 records, respectively (Fig. 5, [22]). After eligibility criteria were applied, 14 original studies were included in this study [2, 5, 10, 12, 13, 18, 25, 27, 29, 33, 39, 41–43], whereof 13 were included in the quantitative analysis [2, 5, 10, 12, 13, 18, 27, 29, 33, 39, 41–43]. Reasons for exclusion of the remaining 13 articles are listed in Fig. 5. Citation tracking did not add any additional study.

### Quality assessment

Quality assessment scores of the included studies are shown in Table 1. All studies were published in a peer-reviewed journal and reported on follow-up time. None of the studies included a control group nor randomization or masked assessment. With an average-quality score of 4.2 (range 3–6), all included studies were scored as low quality. An article was considered low-quality if at least four of the criteria were missing. Quality of evidence was comparable between the different included studies. Results on the best-evidence synthesis are reported in Table 2.

**Table 1:** Quality of evidence

	PRJ	C	R	MA	G	CC	COI	I/E	F/U	Stats	Total
Adachi et al. [2]	+				+				+	+	4
Cho et al. [5]	+				+		+	+	+	+	6
Hui et al. [10]	+				+				+		3
Karlsson et al. [12]	+				+				+		3
Kollias et al. [13]	+						+		+		3
Maffulli et al. [18]	+				+		+		+	+	5
Ogawa et al. [25]	+				+	+			+	+	5
Porter et al. [27]	+				+		+		+		4
Raikin et al. [29]	+				+		+		+	+	4
Saxena et al. [33]	+						+		+	+	5
Tomihara et al. [39]	+				+				+	+	4
Walther et al. [41]	+						+		+		3
Wang et al. [42]	+				+	+			+	+	5
Zhenbo et al. [43]	+				+		+		+	+	5
Total	14				11	2	8	1	14	9	59
Average											4.2

*PRJ* Peer-reviewed journal, *C* control group, *R* randomization, *MA* masked assessment, *G* gender, *CC* comorbid conditions, *COI* conflict of interest, *I/E* inclusion/exclusion, *F/U* follow-up

**Table 2:** Best-evidence synthesis

Outcomes	Outcome measure	High-quality studies	Low-quality studies	BES
Group A: SPR repair	RTS rate		[2, 5, 10, 39]	Moderate
	AOFAS improvement		[2, 5, 18, 39]	Moderate
	Satisfaction		[5, 18]	Moderate
Group B: Groove deepening and SPR repair	RTS rate		[5, 12, 13, 27, 33, 41]	Moderate
	AOFAS improvement		[5, 13, 29, 33, 41]	Moderate
	Satisfaction		[5, 29]	Limited
Group C: Bony procedure	RTS rate		[39, 43]	Limited
	AOFAS improvement		[39, 43]	Limited
	Satisfaction		[43]	Limited
Group D: Rerouting procedure	RTS rate		[42]	Limited
	AOFAS improvement		[42]	Limited
	Satisfaction		[42]	Limited

*BES* best-evidence synthesis

### Evaluation of study characteristics

Of the 14 studies included, 12 were case series [2, 10, 12, 13, 18, 25, 27, 29, 33, 41–43], and two were comparative case series [5, 39]. Baseline characteristics are shown in Table 3. Treatment options were divided into four different groups: group A: SPR repair [1, 5, 10, 18, 39], group B: groove deepening and SPR repair [5, 12, 13, 25, 27, 29, 33, 41], group C: bony procedure [39, 44] and group D: rerouting procedure [42]. Outcomes are shown in Table 4. Statistical analysis was performed with group A and B. Analysis could not be performed on group C and D, as numbers of participants were too small. Characteristics and statistical analysis of the groups are shown in Table 5.

**Table 3:** Baseline characteristics

Study	Study design	Group	Patient demographics	Concomitant ankle comorbidities	Follow-up
Adachi et al. [2]	Retrospective case series	A	*N* = 20, age = 24 year gender M/F = 17/3	Lateral ankle instability (*N* = 2)	Mean = 38 months (24–86 months)
Cho et al. [5]	Prospective, comparative case series	A	*N* = 16 (29), age = 21 year gender M/F = 16/0	*N* = 0	Mean = 33 months (22–45 months)
Cho et al. [5]	Prospective, comparative case series	B	*N* = 13 (29), age = 20 year gender M/F = 13/0	*N* = 0	Mean = 25 months (17–38 months)
Hui et al. [10]	Retrospective case series	A	*N* = 21, age = 24 year gender M/F = 18/3	Not reported	Mean = 112 months (42–180 months)
Karlsson et al. [12]	Retrospective case series	B	*N* = 15, age = 23 year gender M/F = 10/5	Not reported	Mean = 42 months (24–84 months)
Kollias et al. [13]	Retrospective case series	B	*N* = 11, age = 25 year gender M/F = unknown	Intra articular changes (*N* = 10), lateral ankle instability (*N* = 3)	Mean = 72 months (24–102 months)
Maffulli et al. [18]	Retrospective case series	A	*N* = 14, age = 25 year gender M/F = 14/0	Not reported	Mean = 38 months (22–47 months)
Ogawa et al. [25]	Retrospective case series	B	*N* = 15, age = 33 year gender M/F = 8/7	*N* = 0	Mean = 13 months (3–36 months)
Porter et al. [27]	Case series	B	*N* = 13, age = 24 year gender M/F = 9/4	*N* = 0	>12 months
Raikin et al. [29]	Retrospective case series	B	*N* = 14, age = 34 year gender M/F = 14/0	Peroneal brevis rupture (*N* = 5) Peroneal longus rupture (*N* = 1)	Mean = 32 months (26–45 months)
Saxena et al. [33]	Prospective cohort study	B	*N* = 31, age = 33 year gender M/F = unknown	Peroneal brevis rupture (*N* = 9) Ankle instability (*N* = 6)	>2 years
Tomihara et al. [39]	Retrospective, comparative case series	A	*N* = 19 (15 athletes), age = 23 year gender M/F = 15/4	Not reported	Mean = 51 months (18–120 months)
Tomihara et al. [39]	Retrospective, comparative case series	C	*N* = 15 (11 athletes), age = 17 year gender M/F = 10/5	Not reported	Mean = 66 months (18–210 months)
Walther et al. [41]	Case series	B	*N* = 23, age = 34 year gender M/F = unknown	*N* = 0	24 months
Wang et al. [42]	Retrospective case series	D	*N* = 17, age = 23 year gender M/F = 17/0	*N* = 0	Mean = 28 months (24–60 months)
Zhenbo et al. [43]	Retrospective, comparative case series	C	*N* = 26, age = 29 year gender M/F = 18/8	*N* = 0	Mean = 57 months (36–96 months)

*Group A* SPR repair, *Group B* groove deepening and SPR repair, *Group C* bony procedure, *Group D* rerouting procedure

**Table 4:** Outcomes

Study	Group^a^	Return to sports	AOFAS	Satisfaction^b^	Redislocation
Adachi et al. [2]	A	83 %	Pre *m* = 76, post *m* = 93		*N* = 0
Cho et al. [5]	A	100 %, mean 3.0 months	Pre *m* = 60, post *m* = 93	*E* = 4, *G* = 10, *p* = 2	*N* = 1
Cho et al. [5]	B	100 %, mean 3.1 months	Pre *m* = 59, post *m* = 91	*E* = 3, *G* = 9, *p* = 1	*N* = 0
Hui et al. [10]	A	86			*N* = 0
Karlsson et al. [12]	B	100 %, mean 4.5 months			*N* = 0
Kollias et al. [13]	B	91 %, mean 9.1 month	Pre *m* 53, post *m* 96		*N* = 0
Maffulli et al. [18]	A		Pre *m* = 5, post *m* = 95	*E* = 12, *G* = 2	*N* = 0
Ogawa et al. [25]	B		Post *m* = 87		*N* = 0
Porter et al. [27]	B	100 %, mean 3.0 months			*N* = 0
Raikin et al. [29]	B		Pre *m* = 61, post *m* = 93	*E* = 9, *G* = 4, *F* = 1	*N* = 1
Saxena et al. [33]	B	100 %, mean 3.2 months	Pre *m* = 58, post *m* = 97		*N* = 1
Tomihara et al. [39]	A	80 %, mean 2.9 month	Pre *m* = 78, post *m* = 93		*N* = 0
Tomihara et al. [39]	C	54.40 %, mean 3.9 months	Pre *m* = 77, post *m* = 89		*N* = 2
Walther et al. [41]	B	100 %	Pre *m* = 69, post *m* = 95		*N* = 0
Wang et al. [42]	D	100 %, mean 2.8 months	Pre *m* = 73, post *m* = 100	*E* = 17	*N* = 0
Zhenbo et al. [43]	C	88 %, mean 4.4 months	Pre *m* = 56, post *m* = 88	*E* = 12, *G* = 11, *F* = 3	*N* = 0

^a^ *Group A* SPR repair, *Group B* groove deepening and SPR repair, *Group C* bony procedure, *Group D* rerouting procedure

^b^ *E* excellent, *G* good, *F* fair, *P* poor

**Table 5:** Baseline characteristics and outcomes groups

	Group A SPR repair	Group B Groove deepening and SPR repair	*p* value	Group A + B
Number of patients	*N* = 90	*N* = 120		*N* = 210
Gender	M: 76 (88 %), F: 10 (12 %)	M: 61 (71 %), F: 25 (29 %)		M: 137 (80 %), F: 35 (20 %)
^a^	[2, 5, 10, 18, 39]	[5, 25, 27, 29, 33]		[2, 5, 10, 18, 25, 27, 29, 33, 39]
Age	Mean 23 ± 1.5 years	Mean 28 ± 5.8 years	*p* = 0.099	Mean 26 ± 5.1 years
^a^	[2, 5, 10, 18, 39]	[5, 13, 25, 27, 29, 33, 41]		[2, 5, 10, 13, 18, 25, 27, 29, 33, 39, 41]
AOFAS Pre-operative Post-operative Improvement *p* value Improvement	Mean 67 ± 12 Mean 93 ± 0.79 Mean 26 ± 13 *p* = 0.0249	Mean 60 ± 5.6 Mean 94 ± 2.3 Mean 35 ± 6.4 *p* = 0.0003	*p* = 0.24	Mean 63 ± 9.2 Mean 94 ± 1.8 Mean 31 ± 3.3 *p* < 0.0001
^a^	[2, 5, 18, 39]	[5, 13, 29, 33, 41] [2, 4, 8, 9, 11]		[1, 2, 4, 5, 8–11]
RTS rate	Mean 87 ± 8.9 %	Mean 99 ± 3.7 %	*p* = 0.022	Mean 93 ± 8.4 %
^a^	[1–3, 10]	[5, 13, 27, 33, 41]		[2, 5, 10, 13, 27, 33, 41]
RTS time	Mean 3.0 ± 0.070 months	Mean 4.6 ± 2.6 months	*p* = 0.44	Mean 4.1 ± 2.3 months
^a^	[5, 39]	[5, 13, 27, 33]		[5, 13, 27, 33, 39]

^a^Studies reported on this outcome

### Rate of return to sports

Eleven studies reported on RTS rate [2, 5, 10, 12, 13, 27, 33, 39, 41–43]. Two of the studies excluded non-athletes from the RTS analysis [2, 39], leaving a total of 230 evaluated patients. Surgical treatment of peroneal tendon dislocation resulted in a RTS rate from 55 to 100 %. In group A, 83 to 100 %; group B, 91 to 100 %; group C, 55 to 88 %; and group D, 100 % of the patients were able to return to sports. A difference was found between group A and B (*p* = 0.022).

### Time to return to sports

Eight studies reported on time to RTS, with a number of 168 included patients [5, 12, 13, 27, 33, 39, 42, 43]. The time to RTS ranged from 1.2 to 12 months (Table 4). Mean time to return to sports was 3.0 ± 0.070 months in group A and 4.6 ± 2.6 months in group B. Time to return to sports did not differ between groups A and B (*p* = 0.44).

### AOFAS score

Eleven studies used the AOFAS scale as an outcome measure [2, 5, 13, 18, 25, 29, 33, 39, 41–43]. Mean pre-operative AOFAS score ranged from 53 to 78, and mean post-operative AOFAS score ranged from 87 to 100. All studies reported a significant improvement in the AOFAS score after surgical treatment. There was no significant difference in improvement between group A and B (*p* = 0.24).

### Satisfaction

Five studies (100 patients) reported on patient satisfaction [5, 18, 29, 42, 43]. Fifty-seven patients stated that the results were ‘excellent’, and 36 patients evaluated the treatment as ‘good’. ‘Fair’ patient satisfaction was reported in six patients, and one patient evaluated the treatment as ‘poor’. In total, over 90 % of the patients reported a ‘good’ or ‘excellent’ satisfaction.

### Redislocation

All studies reported on redislocation rates [2, 5, 10, 12, 13, 18, 25, 27, 29, 33, 39, 41–43]. In 10 studies, there was no recurrence of peroneal dislocation. Cho et al. [5] reported redislocation in one patient which was treated with SPR repair resurgery. In the study by Tomiharo et al. [39], two patients treated with a bony procedure had post-operative peroneal tendon redislocation. Management of the redislocation was not reported. Saxena et al. [33] and Raikin et al. [29] both reported redislocation in one patient after groove deepening and SPR repair. Neither study reported on the management of the redislocation.

## Discussion

The most important finding of the present study was that both isolated SPR repair and SPR repair combined with a groove-deepening procedure are successful treatment options in the management of peroneal tendon dislocation, with a higher rate of return to sports in patients treated with groove deepening. Since peroneal tendon dislocation is most present in the athletic population [3, 23], surgical treatment with a combination of groove deepening and SPR repair is recommended. However, this finding was based on limited evidence due to a lack of high-quality studies.

In the current study, treatment with SPR repair (group A) and treatment with groove deepening and SPR repair (group B) was compared. Between 1995 and 2015, only three studies reported on bony procedures (group C) and rerouting of the peroneal tendons (group D) [39, 42, 43]. Based on the best-evidence syntheses and the small number of patients, it was concluded that evidence for groups C and D is limited, and therefore, the two groups  were excluded from further analysis. A possible explanation for the lack of studies in groups C and D is the relatively high rate of occurrence of complications including non-union and fractures previously reported, which limited their use in current practice [15, 19–21, 36].

The high return-to-sports rate in both treatment groups A and B (83–100 %) and improvement in the AOFAS score after treatment, provides evidence for good surgical outcomes (*p* < 0.0001). The redislocation rate was less than 1.5 % in both groups, and other major complications were uncommon. As far as reported, over 90 % of the patients were satisfied with their treatment. These findings are confirmed in the only published study which compared groups A and B in a prospective comparative case series [5]. In the current study a higher rate of return to sports was found in patients treated with groove deepening and SPR repair, compared with patients treated with SPR repair alone.

To our knowledge, no previous systematic review has been published addressing the surgical treatment of peroneal tendon dislocation. A review from Oliva et al. [26] demonstrated that reattachment of the SPR is the most appropriate technique when utilizing an anatomic approach. This study, however, was not based on systematic analysis of collected studies and did not provide sufficient data to substructure their conclusions. In the current study, no difference in the time to return to sports was found in patients treated with SPR repair compared with other treatments. In addition, a higher rate of return to sports was found in patients treated with both SPR repair and groove deepening.

Peroneal tendon subluxation has been attributed to forceful ankle dorsiflexion and concomitant reflex peroneal muscle contraction leading to rupture of the SPR and has been associated with anatomic variants including acquired peroneal retinaculum laxity, absence of a groove in the fibula, presence of a convex surface on the posterior aspect of the malleolus, low-lying muscle belly and the presence of a peroneus quartus muscle [14, 32, 37, 42, 43]. Diminished volume within the superior peroneal tunnel may render tendons more prone to dislocation. This volume is determined not only by the fibular shape, but also by the fibrocartilaginous periosteal cushion. In patients with peroneal tendon dislocation, this periosteal cushion is often torn from the fibula, decreasing the volume of the tunnel when only reattaching the SPR. Retromalleolar groove-deepening procedures may provide stabilization of the peroneal tendons behind the lateral malleolus, thereby preventing redislocation [14]. Title et al. [38] reported a cadaveric biomechanical study analysing pressures at different positions of the ankle before and after peroneal groove-deepening procedures. Significant decreases in pressure were noted within the distal and middle groove at all ankle positions after the procedure. Retromalleolar groove deepening with peroneal retinaculum reconstruction resulted in an increased tunnel volume reducing the risk of redislocation, improving both patient rehabilitation and the ability to return to sport.

The current study is not without limitations. First, this systematic review shows that there is a lack of high-quality studies. All studies scored 0 points on the following quality of evidence criteria: control group, randomization and masked assessment. Therefore, caution should be used when making conclusive statements based on this level of quality. Although peroneal tendon dislocation is a relatively rare condition, there has been a large number of treatment techniques described making it difficult to set up a high-level of evidence study [26, 30].

Second, the AOFAS has been used as an outcome measure in the study. The validity of the AOFAS is undetermined. Nevertheless, a systematic review from Hunt et al. showed that the AOFAS score is the most frequently used patient-reported outcome measure in foot and ankle surgery. Given the fact that most of the studies included reported AOFAS outcomes, it was considered that this would be an appropriate measure to compare results of the different studies [11].

A third limitation is the prevalence of lateral ankle comorbidities among patients in some of the included studies, creating risk of selection bias [2, 13, 29, 33]. However, due to the relatively low prevalence of peroneal tendon subluxation, this bias is hard to avoid. In addition, as it is not uncommon that peroneal tendon dislocation occurs with concomitant lateral ankle comorbidities, including these patients creates a more accurate reflection of this patient population.

Another limitation of the study is combining different surgical techniques in the treatment groups. Although the surgical attempts within each group were relatively similar, the specific techniques used varied within each group. Due to small numbers of patients included per study, it was not possible to analyse different surgical techniques. Therefore, combining different techniques in treatment groups was the best option for comparison.

### Future prospects

Future high-level prospective studies are necessary to establish a management algorithm for patients presenting with dislocation of peroneal tendons. Based on the quality of evidence assessment, it is evident that future studies should include control groups, randomization and masked assessment. Peroneal tendon dislocation is most prevalent in the athletic population; therefore, attention should be directed towards return to sports rates and time to return to sports [3, 23].

## Conclusion

Surgical treatment of peroneal tendon dislocation provides good outcomes, high satisfaction and a quick return to sports. A combination of a groove deepening and SPR repair gives a higher rate in return to sports when compared to a SPR repair by itself.
